# Combination of Universal Mechanical Testing Machine with Atomic Force Microscope for Materials Research

**DOI:** 10.1038/srep12998

**Published:** 2015-08-12

**Authors:** Jian Zhong, Dannong He

**Affiliations:** 1 National Engineering Research Center for Nanotechnology, Shanghai 200241, People’s Republic of China; 2 School of Materials Science and Engineering, Shanghai Jiao Tong University, Shanghai 200240, People’s Republic of China

## Abstract

Surface deformation and fracture processes of materials under external force are important for understanding and developing materials. Here, a combined horizontal universal mechanical testing machine (HUMTM)-atomic force microscope (AFM) system is developed by modifying UMTM to combine with AFM and designing a height-adjustable stabilizing apparatus. Then the combined HUMTM-AFM system is evaluated. Finally, as initial demonstrations, it is applied to analyze the relationship among macroscopic mechanical properties, surface nanomorphological changes under external force, and fracture processes of two kinds of representative large scale thin film materials: polymer material with high strain rate (Parafilm) and metal material with low strain rate (aluminum foil). All the results demonstrate the combined HUMTM-AFM system overcomes several disadvantages of current AFM-combined tensile/compression devices including small load force, incapability for large scale specimens, disability for materials with high strain rate, and etc. Therefore, the combined HUMTM-AFM system is a promising tool for materials research in the future.

Surface deformation and fracture processes of materials under external force are important for understanding and developing materials, so it is necessary to develop technology and instruments to investigate surface deformation and fracture processes of materials under external force. In order to analyze surface deformation and fracture processes at micro- and nanometer scale, many tensile/compression devices have been developed and applied to combine with atomic force microscope (AFM)^1^,^2^, scanning electron microscope (SEM)^3^,^4^,^5^, transmission electron microscope (TEM)^6^,^7^,^8^,^9^, and etc. Compared with other electron microscope, AFM is a powerful nanotechnology tool that can be used to reveal the 3D surface structures (topographical image, phase image, etc) of materials under various ambient conditions without pretreatment^10^,^11^,^12^. Therefore, the combination of tensile/compression devices with AFM has attracted more and more attentions for materials research.

Atomic force microscopy (AFM), a type of scanning probe technique, has been successfully applied to image molecules on surface^13^,^14^,^15^, image surface characterization^16^,^17^,^18^,^19^,^20^,^21^,^22^, image biological entities^23^,^24^, manipulate molecules on surface^25^,^26^,^27^,^28^, observe material interactions^29^,^30^,^31^,^32^, analyze molecular force interaction^33^,^34^, study material nanomechnics^35^,^36^,^37^,^38^, and mechanically fabricate 3D nanostructures^39^,^40^,^41^. Nowadays, several custom-designed tensile/compression devices have been developed to be combined with AFM^1^,^2^,^42^,^43^,^44^,^45^,^46^,^47^,^48^,^49^,^50^,^51^,^52^,^53^,^54^. In addition, commercial tensile/compression devices have already been developed by several companies such as Deben UK Limited^55^, Kammrath & Weiss GmbH^56^, and Asylum Research – Oxford Instruments^57^. However, the development and application of these custom-designed and commercial devices are still in their infant stages and have several disadvantages: (i) so far there are no general standard for the fabrication of these devices; (ii) maximum load force of some devices is small; (iii) these devices were miniature and were designed to be mounted onto AFM stage. Therefore, due to their limited working space, they cannot be used to measure large scale specimens (>10 cm in length) following international standards such as ASTM D638-10, ASTM D882-10, ASTM E345-93(2008), and ISO 527-1995; (iv) these devices cannot be used for materials with high strain rate; and (v) until now, the combined tensile/compression device-AFM system has not been applied to explore the relationship among macroscopic mechanical properties, surface nanomorphological changes under external force, and fracture processes of materials.

Currently, commercial universal mechanical testing machines (UMTMs) are widely used to perform tensile measurements of materials for analyzing macroscopic mechanical properties of materials. They can overcome the disadvantages of the custom-designed and commercial tensile/compression devices. Moreover, the combination of UMTM with AFM can be applied to analyze the relationship among macroscopic mechanical properties, the surface nanomorphological changes under external force, and fracture processes of materials. Especially, most materials scientists and engineers are familiar with both UMTM and AFM. Therefore, it is more easily understandable for them to analyze the results from the combined UMTM-AFM system.

Here, we develop a combined horizontal UMTM (HUMTM)-AFM system by modifying UMTM to combine with AFM and designing a height-adjustable stabilizing apparatus. Then the combined HUMTM-AFM system is evaluated. Finally, it is applied for two kinds of typical large scale thin film materials: polymer material with high strain rate (Parafilm M^®^ All-Purpose Laboratory Film) and metal material with low strain rate (aluminum foil) to anlayze their surface deformation and fracture processes.

## Results

### Design and manufacture of the combined HUMTM-AFM system

**Figure 1:**
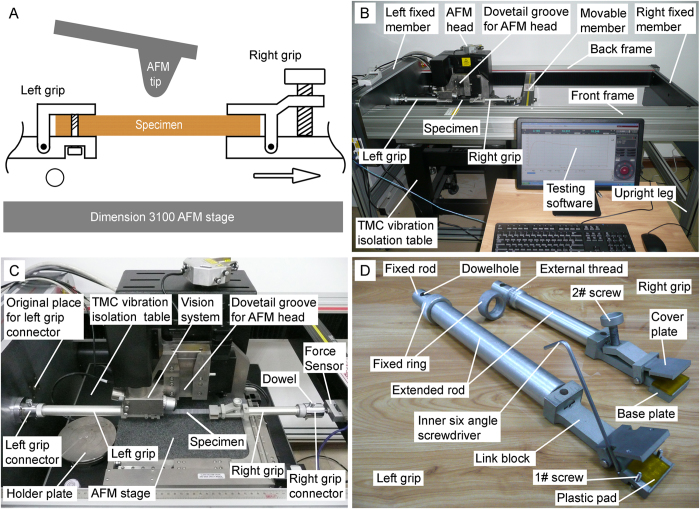
Combined HUMTM-AFM experimental setup. (**A**) The schematic of a test specimen, which was under AFM tip and stretched by HUMTM. The left grip was fixed, as indicated by the hollow circle. The right grip could be moved, as indicated by the hollow arrow. (**B**) Photograph of the combined HUMTM-AFM experimental setup. (**C**) Photograph of a stretched specimen in the combined HUMTM-AFM experimental setup. (**D**) Photograph of the left and right grips.

Generally, the test specimens for DI3100 AFM measurements are horizontally placed on a holder plate. In order to measure the surface nanomorphological changes of a tensiled specimen, the specimen (Fig. 1A) should be placed under AFM tip, held by left and right grips, and tensiled in a horizontal way by UMTM. Therefore, UMTM should be designed and manufactured in a horizontal way to combine with AFM. Then, the experiments can be carried out by moving the right grip to stretch a specimen to a designed displacement and then imaging the surface of the tensiled specimen by AFM.

Here, vertical UMTM (HY0580, Shanghai Hengyi Precision Instruments Co., Ltd) was adapted to HUMTM to combine with AFM (Dimension 3100, Veeco Instruments, USA) by following changes: (i) vertical UMTM was adapted to be parallel to the ground and four upright legs were manufactured to hold up the frames (Fig. 1B); (ii) the HUMTM was placed to be around AFM. The left fixed member and the movable member were located in the left side and the right side of AFM (Fig. 1B), respectively; (iii) the hold plate was removed (Fig. 1C); (iv) new left and right grip connectors were fabricated to make sure the axial line between the grip connectors is under the AFM tip (Fig. 1C); (v) due to the space limitation (the space among vision system, AFM head, and AFM stage) of AFM and long initial work distance between left and right grip connector, custom-designed grips were manufactured as shown in Fig. 1C,D. The left grip is a clip-on grip and the right grip is a leveraged grip. Though the shapes are different, both of them mainly consist of cover plate, base plate, plastic pad, link block, extended rod, fixed ring, external thread, and fixed rod. The plastic pads are sticked on the base plates and the cover plates. The grips can be fixed into the grip connectors by in turn plugging the fixed rods into the grip connectors, plugging the dowels into the dowelholes, and rotating the fixed rings on the external thread to make the fixed rings cling to the grip connectors. By rotating 1# screw in the left grip or rotating 2# screw in the right grip, the plastic pads can be tightened to hold each side of the test specimen.

**Figure 2:**
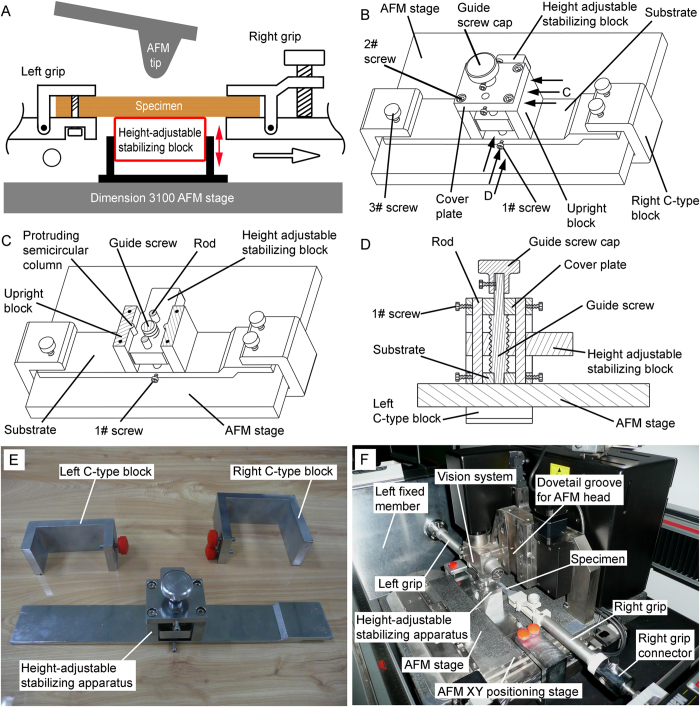
Height-adjustable stabilizing apparatus to minimize ambient vibration. (**A**) The schematic of height-adjustable stabilizing block, which was underneath the test specimen, to minimize ambient vibration. The left grip was fixed, as indicated by the hollow circle. The right grip could be moved, as indicated by the hollow arrow. (**B**) Schematic of height-adjustable stabilizing apparatus on AFM stage. (**C**) Cross-sectional views of height-adjustable stabilizing apparatus on AFM stage taken along arrows C in Fig. 3B. (**D**) Cross-sectional views of height-adjustable stabilizing apparatus on AFM stage taken along arrows D in Fig. 3B. (**E**) Photograph of height-adjustable stabilizing apparatus and C-type blocks. (**F**) Photograph of height-adjustable stabilizing apparatus in the combined HUMTM-AFM experimental setup.

As we know, ambient vibration is a major factor to influence the quality of AFM imaging. Although AFM is located on a TMC vibration isolation table, the HUMTM was located on the ground, which demonstrates the test specimens are not isolated from ambient vibration. In addition, the tensiled thin film specimens acted like a sensor itself^51^, which amplified ambient vibration from HUMTM. Therefore, it is necessary to develop an apparatus to minimize the effect of ambient vibration on specimens. Theoretically, if an apparatus on AFM stage moves a height-adjustable stabilizing block to be underneath a tensiled thin film specimen (Fig. 2A), the stabilized part of specimen can be isolated from ambient vibration, and therefore, AFM observation on this stabilized part of specimen may not be affected by ambient vibration.

In this work, a metal height-adjustable stabilizing apparatus was designed (Fig. 2B–D) and manufactured (Fig. 2E) to move a height-adjustable stabilizing block underneath the thin film specimen (Fig. 2F). The height-adjustable stabilizing apparatus consists of substrate, upright blocks, height-adjustable stabilizing block, rods, guide screw, guide screw cap, cover plate, left C-type block, right C-type block, and three types of screws (1#–3#). The height-adjustable stabilizing block has two concave semicircular column structures, which match to the protruding semicircular column structure of the upright blocks (Fig. 2C). All the cover plate, height-adjustable stabilizing block, and substrate had two corresponding circular through holes, which match the rods. The central places of both the substrate and the cover plate had one corresponding circular through hole, which matches the bottom portion and the upper portion of the guide screw, respectively (Fig. 2D). The height-adjustable stabilizing block has a threaded through hole, which matches the middle threaded portion of the guide screw. Due to the presence of AFM XY positioning stage on AFM stage (Fig. 2F), the substrate was designed to be stair-like and the right side portion is higher than the central portion (Fig. 2B).

### Evaluation of the combined HUMTM-AFM system

Considering that it will take about 10–20 min tensile pause to obtain surface properties by AFM, the effect of the tensile pause on the tensile properties of specimens should be investigated. In addition, it is necessary to evaluate if the height-adjustable stabilizing block can ensure the test specimens are isolated from ambient vibration. Therefore, effect of tensile pause on macroscopic mechanical properties of materials and effectiveness of the height-adjustable stabilizing apparatus for AFM measurements were evaluated prior to intial demonstrations of the combined HUMTM-AFM system.

**Figure 3:**
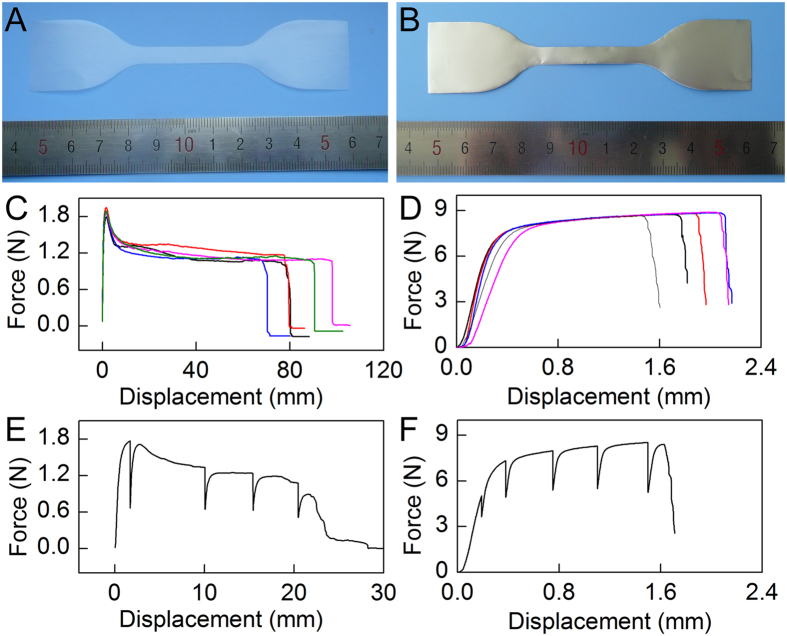
Effect of tensile pause on macroscopic mechanical properties of thin film materials. (**A**) Photograph of Parafilm. (**B**) Photograph of aluminium foil. (**C**) Force-displacement curves of Parafilm specimens without tensile pause by HUMTM. (**D**) Force-displacement curves of aluminium foil specimens without tensile pause by HUMTM. Five test specimens were tested and shown in the figures of (**C**–**D**). (**E**) Typical force-displacement curve of Parafilm specimen with tensile pause of 20 min at displacements of 1.6, 10.0, 15.0, and 20.0 mm. (**F**) Typical force-displacement curve of aluminium foil specimen with tensile pause of 20 min at displacements of 0.18, 0.38, 0.75, 1.11, and 1.52 mm.

In this work, typical polymer thin film material with high strain rate (Parafilm, Fig. 3A) and metal thin film material with low strain rate (aluminum foil, Fig. 3B) were used as test specimens for the evaluation of the combined HUMTM-AFM system.

Firstly, effect of tensile pause on macroscopic mechanical properties of Parafilm specimens was studied. Figure 3C shows the force-displacement curves of Parafilm specimens without tensile pause. The tensiled Parafilm specimens experienced linear elastic zone (steep upward slope) and plastic zone (steep downward slope and gentle downward slope) before break. The maximum force, tensile strength, and elongation at break were 1.87 ± 0.06 N, 2.45 ± 0.07 MPa, and 370% ± 44%, respectively. Figure 3E shows typical force-displacement curve of Parafilm specimen with tensile pause of 20 min at displacements of 1.6, 10.0, 15.0, and 20.0 mm. Inverted force spikes appeared in the curves when the test specimens were continued to be stretched after each pause. The maximum force and tensile strength were 1.76 ± 0.10 N and 2.38 ± 0.07 MPa, respectively. They were similar to those of Parafilm specimens without tensile pause. However, the elongation at break was 147% ± 24%, which was less than that of Parafilm specimens without tensile pause. It demonstrated that tensile pause had no obvious effect on the maximum force and tensile strength of Parafilm, while it decreased the elongation at break of Parafilm. That’s mean, tensile pause had obvious effect on macroscopic mechanical properties of Parafilm. Therefore, in order to correctly analyze the surface property changes, Parafilm test specimens should be only paused once during the measurements.

Secondly, effect of tensile pause on macroscopic mechanical properties of aluminum foil specimens was studied. Figure 3D shows the force-displacement curves of aluminum foil specimens without tensile pause. The tensiled aluminum foil specimens experienced linear elastic zone (steep upward slope) and the plastic zone (gentle upward slope) before break. The maximum force, tensile strength, and elongation at break were 8.79 ± 0.09 N, 104 ± 1 MPa, and 7.76% ± 0.94%, respectively. Figure 3F shows typical force-displacement curve of aluminum foil specimen with tensile pause of 20 min at displacements of 0.18, 0.38, 0.75, 1.11, and 1.52 mm. Inverted force spikes appeared in the curves when the test specimens were continued to be stretched after each pause. The maximum force, tensile strength, and elongation at break were 8.52 ± 0.10 N, 101 ± 1 MPa, and 7.23% ± 0.77%, respectively. They were similar to those of aluminum foil specimens without tensile pause. Therefore, the tensile pause had no obvious effect on macroscopic mechanical properties of aluminum foil, which was different from that of Parafilm. Therefore, multiple tensile pauses can be applied for analyzing the surface nanomorphological changes by AFM.

**Figure 4:**
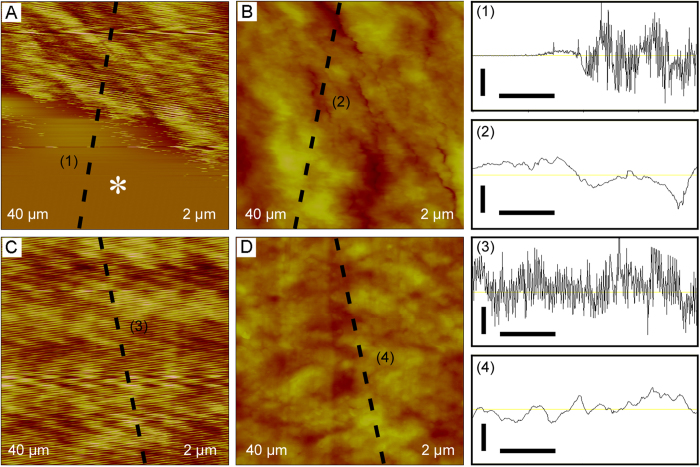
Effectiveness of height-adjustable stabilizing apparatus for AFM measurements. (**A**) AFM height images of Parafilm specimen without the support of height-adjustable stabilizing block. White asterisk indicates untracked surface areas. (**B**) AFM height images of Parafilm specimen with the support of height-adjustable stabilizing block. (**C**) AFM height images of aluminium foil specimen without the support of height-adjustable stabilizing block. (**D**) AFM height images of aluminium foil specimen with the support of height-adjustable stabilizing block. Visual fields and height scales are shown at the lower left corner and the lower right corner, respectively. (1–2) are section analyses along the corresponding white dashed lines from bottom to top in (**A**–**B**). (3–4) are section analyses along the corresponding white dashed lines from top to bottom in (**C**–**D**). The vertical scale bars and horizontal scale bars in (1–4) represent 0.5 and 10 μm, respectively.

Finally, the effectiveness of the height-adjustable stabilizing apparatus for AFM measurements of tensiled thin film materials (Parafilm and aluminum foil) was examined, as shown in Fig. 4. The Parafilm test specimen was stretched to a displacement of 1.6 mm and then was imaged by AFM in turn without (Fig. 4A) and with (Fig. 4B) the support of stabilizing block. Without the support of stabilizing block, the height image showed dense parallel lines (upper part in Fig. 4A) those were resulted from the ambient vibration and flatted area (lower part in Fig. 4A, indicated by white asterisk) that was resulted from the untracked imaging by AFM tip. With the support of stabilizing block, the height image of Parafilm test specimen (Fig. 4B) showed clear micro- and nano-structure characters. These microstructure characters obtained The aluminum foil test specimen was stretched to a displacement of 0.2 mm and then imaged by AFM in turn without (Fig. 4C) and with (Fig. 4D) the support of stabilizing block. Without the support of stabilizing block, the height image (Fig. 4C) showed dense parallel lines those were resulted from the ambient vibration. Compared with Parafilm, no flatted surface was shown in the height image of aluminum foil, which might be resulted from that aluminum foil is more rigid than Parafilm. With the support of stabilizing block, the height image of aluminum foil specimen (Fig. 4D) showed clear micro- and nano-structure characters. Therefore, the use of height-adjustable stabilizing apparatus could minimize the effect of the ambient vibration on AFM measurements and make it is possible to observe the surface properties of tensiled thin film materials.

### Initial demonstrations of the combined HUMTM-AFM system for materials research

As initial demonstrations of the combined HUMTM-AFM system, we investigated the relationship among macroscopic mechanical properties, surface nanomorphological changes under external force, and fracture processes of two kinds of representative large scale thin film materials: polymer material with high strain rate (Parafilm) and metal material with low strain rate (aluminum foil).

**Figure 5:**
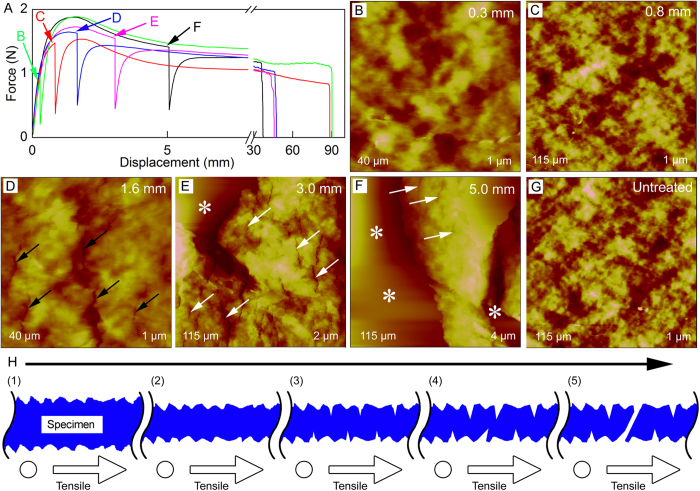
Initial demonstration of the combined HUMTM-AFM system for Parafilm research. (**A**) Representive force-displacement curves of Parafilm test specimens with tensile pause for AFM imaging. The Parafilm specimens were paused at designed displacements of 0.3 (green curve, indicated by green arrow), 0.8 (red curve, indicated by red arrow), 1.6 (blue curve, indicated by blue arrow), 3.0 (magenta curve, indicated by magenta arrow), and 5.0 mm (black curve, indicated by black arrow), separately. (**B**–**G**) AFM height images that are corresponding to tensile displacements in (**A**). Black arrows indicate nanocracks. White arrows indicate small microcracks. White asterisks indicate large microcracks. (**H**) Proposed fracture mechanism of tensiled Parafilm. The left side of Parafilm was fixed, as indicated by the hollow circle. The right side of Parafilm was moved, as indicated by the hollow arrow. See text for details.

Five Parafilm test specimens were stretched to displacement of 0.3, 0.8, 1.6, 3.0, and 5.0 mm, separately. Then the specimens were imaged by AFM with the support of height-adjustable stabilizing block at each displacement. The typical force-displacement curves were shown in Fig. 5A and the corresponding AFM height images were shown in Fig. 5B–F. In contrast, AFM height image of untreated Parafilm was shown in Fig. 5G. No obvious nanomorphological changes or nanocracks were shown in Fig. 5B,C when Parafilm was tensiled in its elastic zone (steep upward slope of green and red force-displacementcurves in Fig. 5A). Nanocracks (indicated by black arrows in Fig. 5D) with a gap width of 508 ± 82 nm appeared when Parafilm was tensiled on the initial position of the plastic zone of Parafilm (steep downward slope of blue force-displacement curve in Fig. 5A). When the tensile displacement was 3.0 mm (middle position of the steep downward slope of magenta force-displacement curve in Fig. 5A), both small microcracks (indicated by white arrows in Fig. 5E) with a gap width of 1825 ± 99 nm and large flat surface areas (indicated by white asterisks in Fig. 5E) with a gap width of several tens of micrometer appeared. The latter was resulted from the untracked imaging by AFM tip.When the tensile displacement was 5.0 mm (bottom position of the steep downward slope of black force-displacement curve in Fig. 5A), compared with AFM image at a tensile displacement of 3.0 mm, the gap width of small microcracks was decreased to 1319 ± 158 nm and large microcracks (flat surface area) with a gap width of several tens of micrometer enlarged (indicated by white asterisks in Fig. 5F). The former might be resulted from the retraction of the area. Therefore, during the tensile process, the fracture mechanism of Parafilm was resulted from that the expansion of nanocracks to microcracks, and finally to fracture of the test specimen.

The elongation at break of Parafilm with tensile pause at 0.3, 0.8, 1.6, 3.0, and 5.0 mm were 371% ± 46%, 358% ± 40%, 214% ± 28%, 186% ± 22%, and 144% ± 18%, respectively. Compared with the elongation at break of Parafilm without tensile pause (Fig. 3C), tensile pause in the elastic zone had no obvious effect on the elongation at break of Parafilm. However, tensile pause in the plastic zone could decrease the elongation at break of Parafilm. AFM results showed that no obvious cracks appeared when Parafilm was tensiled in the elastic zone. However, many nanocracks and microcracks appeared when Parafilm was tensiled in the plastic zone. It demonstrated that tensile pause after the appearance of nanocracks and microcracks might cause the decrease of the elongation at break of Parafilm.

According to above analyses, the possible fracture process of Parafilm was proposed in Fig. 5H. Considering the length of narrow parallel-sided portion increased during the tensile process, the thickness of narrow parallel-sided portion might be decreased and no obivious nanomorphological changes or nanocracked were present when Parafilm was tensiled in its elastic zone (steep upward slope of green and red force-displacementcurves in Fig. 5A), as illustrated in Fig. 5H(1, 2). When the elastic zone switched to the plastic zone, nanocracks appeared, as illustrated in Fig. 5H(3). Then, with the increase of tensile displacements, nanocracks enlarged and converted into microcracks, as illustrated in Fig. 5H(4, 5). Finally, Parafilm was fractured at its break position, as illustrated in Fig. 5H(6).

**Figure 6:**
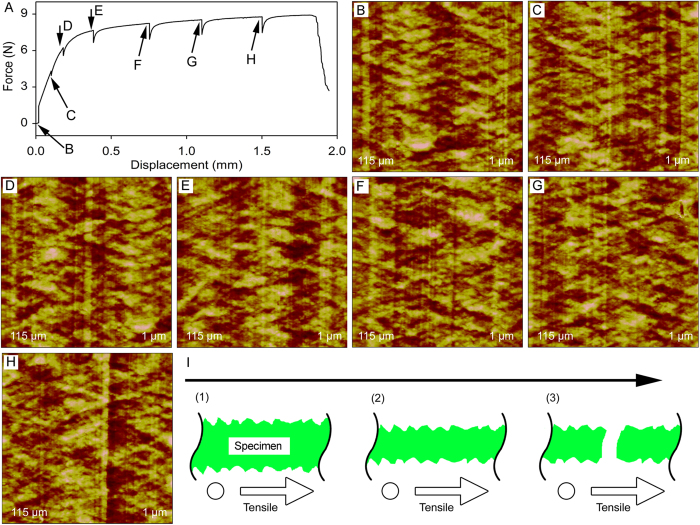
Initial demonstration of the combined HUMTM-AFM system for aluminium foil research. (**A**) Representive force-displacement curve of aluminium foil test specimens with tensile pause for AFM imaging. The specimens were paused at designed displacements of 0.02, 0.10, 0.18, 0.38, 0.75, 1.10, and 1.50 mm, as indicated by black arrows. (**B**–**H**) AFM height images that are corresponding to tensile displacements in (**A**). (**I**) Proposed fracture mechanism of tensiled aluminium foil. The left side of aluminium foil was fixed, as indicated by the hollow circle. The right side of aluminium foil was moved, as indicated by the hollow arrow. See text for details.

Aluminum foil was also chosen as a typical metal specimen with low strain rate. It was tensiled to a designed displacement of 0.02 mm (indicated by arrow B in Fig. 6A) and AFM height image (Fig. 6B) was obtained with the support of height-adjustable stabilizing block. Then the aluminum foil was in turn to be tensiled to different designed displacements (indicated by arrows C–H in Fig. 6A) and imaged by AFM (Fig. 6C–H).

As shown in Fig. 6B–H, no obvious nanomorphological changes or nanocracks were observed by AFM during the tensile process of aluminum foil even after a displacement of about 1.5 mm (about 15% close to the elongation at break). It means no obvious nanocracks appeared when aluminum foil was tensiled in its linear elastic zone and plastic zone of the force-displacement curve, which was different from Parafilm. Considering the length of narrow parallel-sided portion increased during the tensile process, the thickness of narrow parallel-sdied portion might be decreased. Therefore, the possible fracture process was proposed in Fig. 6I. During the tensile process, the aluminum foil was tensiled to be thinner and no obvious nanocracks were shown, as illustrated in Fig. 6I(1, 2). Then the aluminum foil was fractured at its break position after its linear elastic zone and plastic zone, as illustrated in Fig. 6I(3).

## Discussion

Surface deformation and fracture processes of materials under external force are important for understanding and developing materials. Therefore, it is necessary to develop technology and instruments to analyze the surface deformation and fracture processes at micro and nanometer scale. This work focused on the design, manufacture, evaluation, and demonstrations of a combined HUMTM-AFM system. We developed a combined HUMTM-AFM system by modifying UMTM to combine with AFM and manufacturing a height-adjustable stabilizing apparatus. Then the combined HUMTM-AFM system was evaluated. Finally, as initial demonstrations, it was applied for two kinds of typical large scale thin film materials: polymer material with high strain rate (Parafilm M^®^ All-Purpose Laboratory Film) and metal material with low strain rate (aluminum foil) to anlayze their surface deformation and fracture processes.

Compared with current AFM-combined tensile devices, the combined HUMTM-AFM system has several advantages for materials research (Table 1): (i) the HUMTM was adapted from commercially developed vertical UMTM. It is easier for most materials scientists and engineers to undersand the results from the combined HUMTM-AFM system. Therefore, it is easy to build general standard for the development and application of the combined HUMTM-AFM system; (ii) Maximum load force can be adjusted by chosing different force sensor with different force range for HUMTM; (iii) the combined HUMTM-AFM system can be applied for large scale materials (>10 cm long) following international standards. Current most AFM-combined tensile devices were designed to be mounted onto AFM stage and therefore they cannot be used to measure large scale specimens. For example, maximum 3D sizes of specimens for Bamberg, *et al*.’s tensile device were 2 mm thick, 8 mm wide, and 100 mm long^51^. (iv) the combined HUMTM-AFM system can be applied for materials (especially large scale materials) with high strain rate or low strain rate. Current AFM-combined tensile devices were designed to be mounted onto AFM stage and therefore they cannot be used to measure large scale materials with high strain rate. For example, the maximum strain of Bamberg, *et al*.’s tensile device was 500% for a 25 mm long sample. However, the maximum strain of Bamberg, *et al*.’s tensile device would be much lower for a large scale materials (10 cm long); (v) Current AFM-combined tensile devices were almost designed and manufactured by mechanical engineers. However, the design and manufacture of the combined HUMTM-AFM system including the metal height-adjustable stabilizing apparatus are easy to understand for materials scientists and engineers. They can design and optimize the system for their research purpose and manufacture the system with the help of commercial UMTM company; and (vi) the combined HUMTM-AFM system is a useful tool for analyzing the relationship among macroscopic mechanical properties, the surface nanomorphological changes under external force, and fracture processes of materials. Therefore, the combination of UMTM with AFM is an authoritative and simple strategy for materials research.

In order to minimize ambient vibration during AFM imaging, different height-adjustable stabilizing apparatuses were designed and manufactured for current tensile devices and the HUMTM-AFM system. Bhushan *et al*. designed an aluminum block with a smooth curved top surface with a radius of curvature of 25.4 mm and a simple flexible hinge to move the aluminum block up or down^46^,^49^,^50^. The curved top surface of the aluminum block may make it is difficult to engage the AFM tip onto the sample surface on the middle of the curved top surface. Moreover, the simple flexible hinge may bring new vibration. Bamberg *et al*. designed a complexed flexure-based support with a magnetic base to connect to AFM stage^51^. The maximum vertical adjustment was low (1.5 mm), which brought operating difficulites The adjustable support surface was not level when it was lifted, which made the AFM imaging area of the test specimen was not level, and therefore AFM imaging results might not be accurate. Asylum Research – Oxford Instruments designed a height-adjustable pillar with a straight prism to move the pillar to be underneath the test specimen^57^. The pillar was not stationary onto the prism during AFM imaging and might bring new vibration. Compared with these height-adjustable stabilizing apparatuses, the apparatus in this work was designed to improve these weaknesses as mentioned above. The height adjustable stabilizing block was level whenever it was underneath the test specimen or not. The maximum vertical adjustment could be 1 cm, which made that operation easier. After the height adjustable stabilizing block was moved to be underneath the test specimen, by rotating 1# screws, the press on the block from two rods and two protruding semicircular columns made the block stationary, and therefore, no new vibration was brought. The effectiveness experiments of the height-adjustable stabilizing apparatus for AFM measurements of tensiled thin film materials (Fig. 4) confirmed that the height-adjustable stabilizing apparatus could minimize the effect of the ambient vibration.

In this work, the HUMTM-AFM system was applied to investigate the relationship among macroscopic mechanical properties (tensile force-displacement curves), surface nanomorphological changes under external force, and fracture processes of two kinds of representative large scale thin film materials: polymer material with high strain rate (Parafilm) and metal material with low strain rate (aluminum foil). According to the results and analyses, the possible fracture process of Parafilm and aluminum foil were proposed in Fig. 5H and Fig. 6I, respectively. For the Parafilm, the thickness of narrow parallel-sided portion might be decreased and no obivious nanomorphological changes or nanocracked were present when Parafilm was tensiled in its elastic zone (steep upward slope of green and red force-displacementcurves in Fig. 5A). When the elastic zone switched to the plastic zone (steep downward slope of blue force-displacement curve in Fig. 5A) nanocracks appeared, and then the nanocracks enlarged and converted into microcracks after continuous tensile. Finally, Parafilm was fractured at its break position. The aluminum foil was tensiled to be thinner and no obvious nanocracks were present during the tensile process, and then the aluminum foil was fractured at its break position after its linear elastic zone and plastic zone (Fig. 6A). To the best of our knowledge, no similar demonstrations of the relationship among macroscopic mechanical properties (tensile force-displacement curves), surface nanomorphological changes under external force, and fracture processes *of materials* were published until now. However, it should be noted that only the fracture processes of materials were observed by AFM. Further work is necessary to investigate the fracture mechanisms of the materials and compare with classic fracture theories such as dislocations^58^.

We anticipate that this combined HUMTM-AFM system will be widely used for materials research in the future. Potential objective materials of study include almost all the inorganic, organic, and biological materials. Potential applications of the combined HUMTM-AFM system include observing surface nanomorphological changes of materials under external force, probing fracture behaviors of materials under external force, and analyzing the relationship among macroscopic mechanical properties, the surface nanomorphological changes under external force, and fracture processes of materials. It should be noted that AFM tip does not trace the same position during the tensile and imaging test because only right grip was moved. The HUMTM-AFM system can be used to analyze the homogenous materials such as Parafilm M^®^ All-Purpose Laboratory Film and aluminum foil in this work very well because the ex situ imaging area can be thought as the representive morphological changes of these materials during the tensil process. However, some important information might be missing when the HUMTM-AFM system is applied to study non-homogenous materials. Therefore, further work should be necessary to revise the HUMTM-AFM system to make sure AFM tip trace the same position during the tensile and imaging process.

## Methods

### Test specimens preparation

Parafilm M^®^ All-Purpose Laboratory Film with a thickness of 0.127 mm and aluminum foil with a thickness of 0.014 mm were bought from Bemis Co., Inc. (USA) and Shanghai Cleanwrap Co., Ltd. (China), respectively. Test specimens were made by cutting these materials into dumb-bell-shaped “type 5” specimens, which was described in ISO 527-3: 1995, by a customed-designed cutter. These test specimens had a gage length of 25 mm and a narrow section width of 6 mm.

### Tensile measurements by HUMTM

The tensile properties of the thin film specimens were measured using the custom-adapted HUMTM. Each Dumb-bell-shaped “type 5” specimen had an initial distance between grips of 80 mm. The test speeds for Parafilm and aluminum foil test specimens were 5 mm/min and 0.1 mm/min, respectively. All tests were conducted under ambient condition (about 25 °C and 60% relative humility). Five parallel specimens of each material were examined. Tensile experiments by HUMTM were done as follows. Firstly, the left grip was tightened to hold the left side of the test specimen. Secondly, the left grip with the test specimen was fixed into the left grip connector on left fixed member of HUMTM. Thirdly, the right grip was fixed into the right grip connector on movable member of HUMTM. Fourthly, the right grip was tightened to hold the right side of the test specimen. Finally, the test specimens were tensiled and the test data were recorded by HUMTM.

### Assembly of height-adjustable stabilizing apparatus

The height-adjustable stabilizing apparatus was assembled as follows. Firstly, two upright blocks were fixed on the substrate by rotating 2# screws. Secondly, the height-adjustable stabilizing block, 2 rods, and the guide screw were assembled together and placed between the two upright blocks. The bottom portions of the rods and the guide screw were inserted into the substrate. Thirdly, the cover plate was fixed on the two upright blocks. The upper portions of the rods and the guide screw were inserted into the cover plate. Fourthly, the rods were fixed by rotating four 1# screws to go through the substrate/cover plate, which has corresponding threaded through holes, to touch the rods. Fifthly, the guide screw cap was fixed on the upper portion of the guide screw by tightening 1# screw. Finally, both the left side and the right side of the substrate were fixed on AFM stage by using C-type blocks and 3# screws.

### AFM imaging and analysis

Standard AFM contact mode on a Dimension 3100 AFM (Veeco Instruments, USA) was applied in this work. All the experiments were under ambient conditions. NP-series silicon nitride cantilevers with a nominal spring constant of 0.58 N/m were used. AFM scan rate was 1.5 Hz and imaging resolution was 512 × 512 pixel. All images were treatment with “flatten” function using Nanoscope III software (version 5.31r1, Veeco Instruments) prior to analysis.

### Application of the combined HUMTM-AFM system for thin film material research

The application of HUMTM-AFM system for thin film material research could be done as follows. Firstly, the test specimen was held by the left and right grips. Secondly, the height-adjustable stabilizing apparatus was mounted onto the AFM stage and the height-adjustable stabilizing block was under the test specimen. Thirdly, the test specimen was stretched to a designed displacement and the tensile measurement was paused. Fourthly, the height-adjustable stabilizing block was adjusted to be underneath the test specimen by rotating guide screw cap to support the test specimen. Fifthly, AFM tip approached the specimen surface area that was supported by height-adjustable stabilizing block and obtained the surface properties. Finally, the height-adjustable stabilizing block could be lower or not and then repeat step 3 and 5 to obtain the surface properties of tensiled specimens at a new displacement. Because only right grip was moved, the specimens were tensiled to the right direction and the AFM imaging area were different among every position. Three parallel specimens of each material were examined.

### Statistical analysis

All data were presented as the mean value ± standard deviation (SD) for each sample.

## Additional Information

**How to cite this article**: Zhong, J. and He, D. Combination of Universal Mechanical Testing Machine with Atomic Force Microscope for Materials Research. *Sci. Rep*. **5**, 12998; doi: 10.1038/srep12998 (2015).

## Figures and Tables

**Table 1:** Comparisons of custom-designed tensile devices, commercial tensile devices, and the HUMTM-AFM system.

Authors/Company	Specimen size	Actuation	Force sensor	height-adjustable stabilizing apparatus	Maximum tensile displacement	Device size
Aboulfaraj *et al*.^42^	Large scale specimen: Typically, 300 × 200 × 15 mm	High-torque, d.c.-voltage motor	No	No	No specific data. But we can conclude that it can not be high because the device is located on AFM stage.	Miniature, located on AFM stage
Hild *et al*.^43^,^47^	Small scale specimen: typically, maximum sampel size: 17 mm × 10 mm and a height of 10 mm	Screw	No	No	7 mm	Miniature, located on AFM stage
Coupeau *et al*.^2^,^44^	Small scale specimen: typically, nominally 2.5 mm × 2.5 mm × 5 mm	Piezoelectric translators	No	No	120 μm	Miniature, located on AFM stage
Nishino *et al*.^45^	Small scale specimen: typically, a small rectangular sample (width 5 mm × initial length 20 mm)	Dead load	Load cell	No	Dozens of mm	Miniature, located on AFM stage
Bhushan *et al*.^46^,^49^,^50^	Small scale specimen: typically, 38.1 mm long, 6.35 mm wide, and 6–14 μm thick	Stepper motor	Beam-type strain gauge force sensor with a stiffness of 18kN/m	Yes	10 mm	Miniature, located on AFM stage
Chasiotis *et al*.^48^	Small scale specimen: typically, 400 μm long with 50 × 2 μm cross section	Inchworm	Load cell: 0.5N	No	No specific data. But we can conclude that it can not be high because the device is located on AFM stage.	Miniature, located on AFM stage
Bamberg *et al*.^51^	Small scale specimen: Maximum sample size: 2 mm thick, 8 mm wide, 100 mm long; Minimum sample size: 10 μm thick, 1 mm wide, 20 mm long.	Stepper motor	Load cells: 25N, 250N, 4.4 kN	Yes	125 mm	Miniature, located on AFM stage
Isono *et al*.^52^	Small scale specimen: typically, 3 mm long, 0.3 mm wide, and 19 μm thick	PZT actuator	Load cell with a resolution of 0.38 μN	No	No specific data. But we can conclude that it can not be high because the device is located on AFM stage.	Miniature, located on AFM stage
Thomas *et al*.^53^	Small scale specimen: typically, dumbbell-shaped samples with gauge length 24 mm and width 5 mm	Stepper motor	No specific data.	No	No specific data. But we can conclude that it can not be high because the device is located on AFM stage.	Miniature, located on AFM stage
Lang *et al*.^1^,^54^	Small scale specimen: typically, dumbbell-shaped samples with full length 3.5 mm and width 0.75 mm	Piezo actuator	Foce sensing beam (maximum detectable force of 60N)	No	Dozens of μm	Miniature, located on AFM stage
Deben UK Limited^55^	Small scale specimen: 10–20 mm long or 25–35 mm long	Piezo motor	Load cells: 200N, 2kN,5kN	No	10 mm, optional 20 mm	Miniature, located on AFM stage (110 mm × 58 mm × 33 mm)
Kammrath & Weiss GmbH^56^	Small scale specimen: 28–60 mm long, 10 mm wide maximum, 3 mm thick maximum.	Stepper motor	Load cells: 10N, 20N, 50N, 100N, 200N, 500N, 1kN, 2kN, 5kN, 10kN	No	5 mm	Miniature, located on AFM stage (150 mm × 55 mm × 220 mm)
	Should have reamed holes at both ends (d = 4 mm) for two hardened precision dowel pins.					
Asylum Research–Oxford Instruments^57^	Small scale specimen: 12 mm wide maximum, 41 mm long minimum, 6 mm thick maximu	Sample positioning knob	Force sensors: 20N, 80N	Yes	120 mm (30 mm relaxed to 150 mm fully stretched)	Miniature, located on AFM stage
HUMTM-AFM system	Large scale specimen: Maximum sample size: 5 mm thick, dozen of mm wide, dozen of cm long; minimum sample size: 10 μm thick, 1 mm wide, 20 mm long	Stepper motor	Load cells: 10N, 20N, 50N, 100N, 200N, 500N, 1kN, 2kN, 5kN, 10kN	Yes	Generally more than 100 cm maximum	Large scale, around AFM
